# A Rare Entity: Bilateral First Rib Fractures Accompanying Bilateral Scapular Fractures

**DOI:** 10.1155/2015/428640

**Published:** 2015-06-14

**Authors:** Gultekin Gulbahar, Tevfik Kaplan, Hasan Bozkurt Turker, Ahmet Gokhan Gundogdu, Serdar Han

**Affiliations:** ^1^Division of Thoracic Surgery, Dr. Nafiz Korez Sincan State Hospital, Ankara, Turkey; ^2^Department of Thoracic Surgery, Ufuk University School of Medicine, Ankara, Turkey; ^3^Division of Orthopedics and Traumatology, Dr. Nafiz Korez Sincan State Hospital, Ankara, Turkey; ^4^Division of Thoracic Surgery, Arnavutkoy State Hospital, Istanbul, Turkey

## Abstract

First rib fractures are scarce due to their well-protected anatomic locations. Bilateral first rib fractures accompanying bilateral scapular fractures are very rare, although they may be together with scapular and clavicular fractures. According to our knowledge, no case of bilateral first rib fractures accompanying bilateral scapular fractures has been reported, so we herein discussed the diagnosis, treatment, and complications of bone fractures due to thoracic trauma in bias of this rare entity.

## 1. Introduction

Rib fractures commonly occur following blunt thoracic trauma and they are seen more frequently in the lower thoracic cage, where the ribs are relatively less protected. First rib fractures are scarce due to their well-protected anatomic locations. Generally, they occur following blunt traumas of high energy [1]. But indirect trauma, sudden contraction of the neck muscles, stress, and fatigue fractures may also give rise to this rare entity [2]. Close and cautious follow-up is necessary for early and late complications, because of the neighboring brachial plexus and the vascular structures. Similarly bilateral scapular fractures are rare and are related to high energy trauma [3]. While the treatment option for noncomplicated first rib fractures is conservative, some certain scapular fractures may necessitate surgical intervention. First rib fractures may be together with scapular or clavicular fractures but on the other hand the presence of these fractures together is very rare. Herein we report a case with bilateral first rib fracture accompanying bilateral scapular fracture treated nonsurgically.

## 2. Case

**Figure 1:**
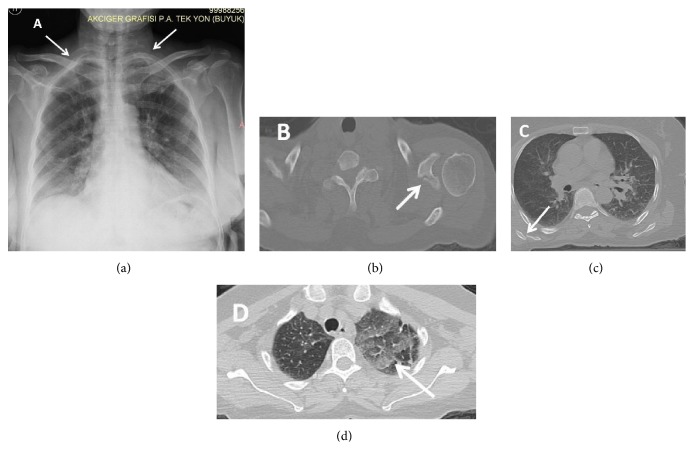
(a) Bilateral first rib fractures (white arrows). (b) Left scapular glenoid fracture (white arrow). (c) Right scapular body fracture (white arrow). (d) Pulmonary contusion (white arrow).

A seventy-seven-year-old female patient was admitted to the emergency department because of a motor vehicle accident. The patient was accepted to the trauma care unit. She was in good state of health, conscious with a Glasgow Coma Scale score of 15, and her hemodynamic state was stable. On physical examination, she had bilateral painful shoulder movements, bilateral tenderness of the lateral hemithoraces, tenderness on the back, and a 7 cm laceration of the scalp on the left parietal region. The hematological and biochemical blood analyses were consistent with the trauma. Bilateral first rib fractures were detected on chest X-ray (Figure 1(a)). Additionally computed axial tomography (CAT) of the chest revealed bilateral scapular fractures and bilateral limited pulmonary contusions in both hemithoraces (Figures 1(b), 1(c), and 1(d)). No neurovascular deficits were detected on the physical and radiologic examinations of the upper extremities. According to bone mineral densitometry the patient did not have osteoporosis. Conservative treatment was initiated in the intensive care unit. During the follow-up, the symptoms subsided and there was no complication. Thus, the patient was discharged with analgesics, bed rest, and bilateral body fixation for scapular fractures on the seventh day of the trauma.

## 3. Discussion

Fractures of the scapula and the first rib are quite rare [1–4]. The fractures of the first rib require injuries of high energy due to its profound location and good protection by the overlying soft tissue, clavicle, and scapula [3]. Similarly fractures of the scapula are scarce and constitute only about 1% of all fractures [4]. Although scapular and first rib fractures commonly occur with severe blunt trauma, first rib fractures can also be caused by sudden and powerful contraction of the muscles of the neck and stress fractures can also be seen [3, 5]. Scapular fractures can also develop due to muscle spasms, electrical shock, epileptic seizures, and metabolic imbalance [2].

In cases of high energy trauma caused first rib fractures, fatal complications of the neighboring structures such as subclavian vasculature, brachial plexus, and mediastinal contents. Early complications of the first rib fractures are pneumothorax, rupture of apex of lung, Horner's syndrome, injury of the brachial plexus, injury of the subclavian artery, pleurisy, trachea-esophageal fistula formation, aneurysm of the aortic arch, and abscess formation in the clavicular neighborhood [2]. Such complications are more common with unilateral fractures rather than bilateral ones. Thus, first rib fracture cases require immediate medical attention to evaluate the accompanying life-threatening injuries [1]. On physical examination, pulse deficit of the upper extremity, blood pressure difference between the upper and lower extremities, edema of the extremity, and motor and sensory neurological deficits must be carefully sought and CAT, magnetic resonance imaging, and electromyography studies must be applied in case of suspicion. In cases without neurovascular injuries, the choice of treatment is analgesia, bed rest, and hot compression. Late complications of the first rib fracture cases are thoracic outlet syndrome and nonunion [2].

Since the scapular fractures are related to high energy traumas like the first rib fractures, multiple system traumas may accompany the situation [3]. So the patients with the scapular fractures must be examined thoroughly starting with the thoracic cage. In physical examination of the scapula, local tenderness, swelling, and painful shoulder movements may be observed. The deformation of the scapula may lead to hematoma formation and rotator cuff injury, which is characterized by the weakness in the movement of shoulder joint [5].

High quality X-ray and CAT are preferred for the diagnosis of scapular fracture [4]. Three-dimensional reconstructions of CAT images may be helpful in detecting the exact type of fracture but it may be insufficient in the diagnosis of nondisplaced fractures. Thus, it must be used along with CAT scan [6]. Such diagnostic procedures are also needed for deciding the modality of treatment. A displacement of greater than 5 millimeters of the glenoid fossa, greater than 10 mm displacement of the glenoid rim, disrupted superior shoulder suspensor complex, greater than 1 centimeter translation or greater than 40° angulations of the glenoid neck favor surgical treatment [4, 7, 8]. In scapular fracture cases which do not require surgery, bilateral shoulder joint immobilization is the treatment option [4].

In our case, the diagnosis was made through X-ray and CAT studies. Right-sided scapular body fracture and left-sided glenoid fracture were found to accompany bilateral rib fractures. No neurovascular or mediastinal complication was seen. Analgesics, hot compression, and bilateral shoulder joint immobilization were used for nonsurgical treatment. Immobilization was discontinued after three weeks. At the end of one-month follow-up, there was a significant decrease in the amount of pain.

## 4. Conclusion

Bilateral first rib fractures accompanying bilateral scapular fractures are quite rare and according to our knowledge our case is the single one in English medical literature. Although isolated bilateral scapular fracture and isolated bilateral first rib fractures may be related to indirect trauma, our case, which contains both entities, is related to high energy trauma. First rib fractures must be overinvestigated in the emergency department because of the probability of major vascular and neurological injuries that may accompany them.
